# Molecular cytogenetic study of the European bitterling *Rhodeus amarus* (Teleostei: Cyprinidae: Acheilognathinae)

**DOI:** 10.1007/s10709-014-9761-x

**Published:** 2014-03-28

**Authors:** Lech Kirtiklis, Konrad Ocalewicz, Marzena Wiechowska, Alicja Boroń, Piotr Hliwa

**Affiliations:** 1 Department of Zoology, Faculty of Biology and Biotechnology, University of Warmia and Mazury, 10-718 Olsztyn, Poland; 2 Department of Marine Biology and Ecology, Institute of Oceanography, University of Gdansk, 81-378 Gdynia, Poland; 3 Department of Ichthyology, Faculty of Environmental Sciences, University of Warmia and Mazury, 10-718 Olsztyn, Poland

**Keywords:** Bitterling, Chromosome banding, Endangered cyprinids, FISH, PRINS, rDNA

## Abstract

The European bitterlings (*Rhodeus amarus*) from the Eastern locations were cytogenetically examined by conventional and molecular techniques. All analyzed individuals presented invariably the same chromosomal constitution of 2n = 48, with 8 metacentrics + 20 submetacentrics + 20 subtelo-acrocentrics and C-banding positive heterochromatin at the pericentromeric regions in most of the chromosomes. Moreover, some of the chromosomes had short arms entirely built with heterochromatin. GC-rich Ag-NORs (nucleolus organizer regions) were located at the short arms of two submetacentric chromosomes, and the length polymorphism of these regions was found. Multiple location of 28S rDNA sequences with fluorescence in situ hybridization signals was observed on the long and/or short arms of three submetacentric chromosomes including NOR regions and short arms of three to five acrocentric chromosomes in the studied fish. 5S rDNA sites were found on the short arms of two subtelocentric chromosomes, and telomeric repeats were localized at the ends of all chromosomes. Provided results have expanded our knowledge concerning genetic characteristics of the European bitterlings that may be profitable in the conservation programs of this endangered species.

## Introduction

The European bitterling (*Rhodeus amarus* Bloch, 1782) is a small freshwater cyprinid fish belonging to the subfamily Acheilognathinae, a group including approximately 73 species inhabiting Eurasian lakes, ponds and slow flowing rivers (Froese and Pauly 2011). The European bitterlings like other bitterling species exhibit an extraordinary reproductive behavior. They display remarkable morphological, physiological and behavioral adaptations for using unionid mussels (Unionidae) as incubators for their eggs during the spawning time (Smith et al. 2004). Bitterling females develop long ovipositors that they use to deposit their eggs into the gills of a mussel through an exhalant siphon. Males fertilize the eggs by releasing sperm into the inhalant siphon of the bivalve, and the incoming water transfers spermatozoa to the eggs. Embryos reside inside the mussel about 1 month until they develop into actively swimming larvae and then leave the mussels.

The European bitterlings show the most western distribution among the bitterlings and are observed in many locations through Europe and Asia Minor (Bryja et al. 2010). Results of the molecular surveys indicate that *R. amarus* sensu stricto is divided into the western lineage (distributed through the Danube River basin and much of Western Europe) and the eastern lineage (occurs in the Carpathians, the Vistula River and most of the East Europe including Eastern part of Poland) (Bohlen et al. 2006; Bryja et al. 2010).

Although abundant in most of its range, *R. amarus* is increasingly threatened by the anthropogenic changes in the natural water reservoirs leading to the water pollution and rapid declining of the unionid fauna (Watters 1996; White et al. 1996). Many European waters, including those inhabited by *R. amarus*, are colonized by an invasive Asian mussel *Anodonta woodiana*. Asian mussels, together with other native mussels, are used by bitterlings for the oviposition. Reichard et al. (2007) reported that *R. amarus* embryos failed to accomplish their embryonic development in *A. woodiana*, because the mussels ejected them outside. This may suggest direct danger for the European bitterling reproduction connected with *A. woodiana* expansion in Europe. Thus, European bitterling has been listed as protected species in some parts of Europe.

As cytogenetic characteristics of the fish populations and lineages may be important tool in wildlife conservation programs, the main goal of the present work was to provide detailed chromosomal information regarding European bitterlings from the eastern locations.

## Materials and methods

**Fig. 1:**
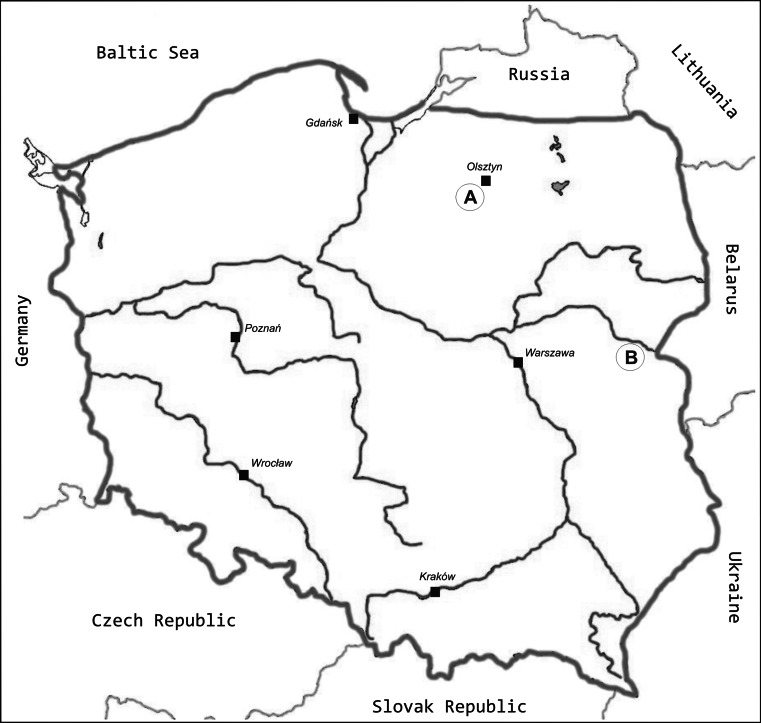
Places of the fish sampling indicated by *uppercase letters in the circle* (*A*—the Starodworskie and the Kortowskie Lakes, *B*—the Bug River)

In the present study we performed cytogenetic analysis on 25 bitterling specimens (8 males and 17 females) from the three natural populations of the Starodworskie Lake (N:53°44′51″, E:20°27′10″), the Kortowskie Lake (N:53°45′41″, E:20°26′42″) and the Bug River near Serpelice village (N:52°16′30″, E:23°03′11″) (Poland) (Fig. 1).

All manipulations and the experimental procedures were provided according to the positive opinion No. 14/2003/N of the Local Ethical Commission from The University of Warmia and Mazury in Olsztyn, Poland.

Taxonomic status of all bitterling individuals were established based on their external morphological features according to Kottelat and Freyhof (2007).

Metaphase spreads were made from the cephalic kidney according to the method described by Ráb and Roth (1988). The karyotype constitution was determined by conventional 5 % Giemsa solution staining. Constitutive heterochromatic regions were visualized in the course of C-banding technique described by Sumner (1972). The nucleolus organizer regions (NORs) were stained by the silver nitrate technique (Ag-NOR) (Howell and Black 1980). The fluorochrome staining chromomycin A_3_ (CMA_3_) was used for detection of the GC- rich regions (Sola et al. 1992).

Fluorescence in situ hybridization (FISH) with human 28S rDNA sequences as a probe (Fujiwara et al. 1998) after labeling with biotin-16-dUTP by nick translation (Roche, Basel, Switzerland) was performed with RNase-pretreated and formamide-denaturated chromosome slides, followed by hybridization with 150 ng of the rDNA probe, per slide. Subsequent to post-hybridization washing (37 °C, 20 min), chromosome slides were subjected to the detection with avidin-FITC (Roche, Basel, Switzerland).

For the chromosomal localization of 5S rDNA sequences, Rhodamine primed in situ labeling (PRINS) kit (Roche, Basel, Switzerland) and a set of primers (5S rev—5′-TACGCCCGATCTCGTCCGATC-3′ and 5S for—5′–CAGGCTGGTATGGCCGTAAGC-3′) facilitating amplification of 5SrDNA sequences were used (Martins and Galetti 1999). PRINS reaction was carried out according to Ocalewicz and Babiak (2003).

Telomeric DNA repeats were detected by FISH using a telomere peptide nucleic acid (PNA) probe conjugated with FITC (DAKO, Denmark) (Ocalewicz and Sapota 2011). Chromosomal DNA was denatured at 84 ^°^C for 3 min under the coverslip in the presence of the PNA probe. Hybridization took place in the darkness at room temperature for 60 min.

For counterstaining, chromosomes after PRINS and PNA-FISH were mounted in 25 μl of antifade reagent (Vectashield) containing DAPI (4′, 6-diamidino-2-phenylindole) (Vector, Burlingame, USA).

### Microscopy processing

At least 15–20 metaphase spreads from each individual were studied using a Nikon Eclipse 90i fluorescence microscope equipped with ProgRes MFcool camera (Jenoptic, Jena, Germany) and a Zeiss Axio Imager.A1 fluorescence microscope equipped with a fluorescent lamp and a digital camera (Applied Spectral Imaging, Galilee, Israel). Both microscopes were supported by appropriate filter set for the multicolor FISH technique. A Lucia software ver. 2.0 (Laboratory Imaging, Prague, Czech Republic) and Band View/FISH View software (Applied Spectral Imaging, Galilee, Israel) were also used for the capturing and the electronic processing of the images. Post-processing elaboration of all the figures were made using CorelDRAW^®^ Graphics Suite 11 (Corel Corporation, Ottawa, Canada). All the chromosomes were classified according to Levan et al. (1964), where metacentric (m) and submetacentric (sm) chromosomes were considered as bi-armed, while subtelocentric (st) and acrocentric (a) chromosomes as uni-armed. A total of 423 chromosome metaphase spreads were investigated in the study.

Voucher specimens have been preserved frozen at the Department of Zoology and Department of Ichthyology, University of Warmia and Mazury in Olsztyn, Poland.

## Results

**Fig. 2:**
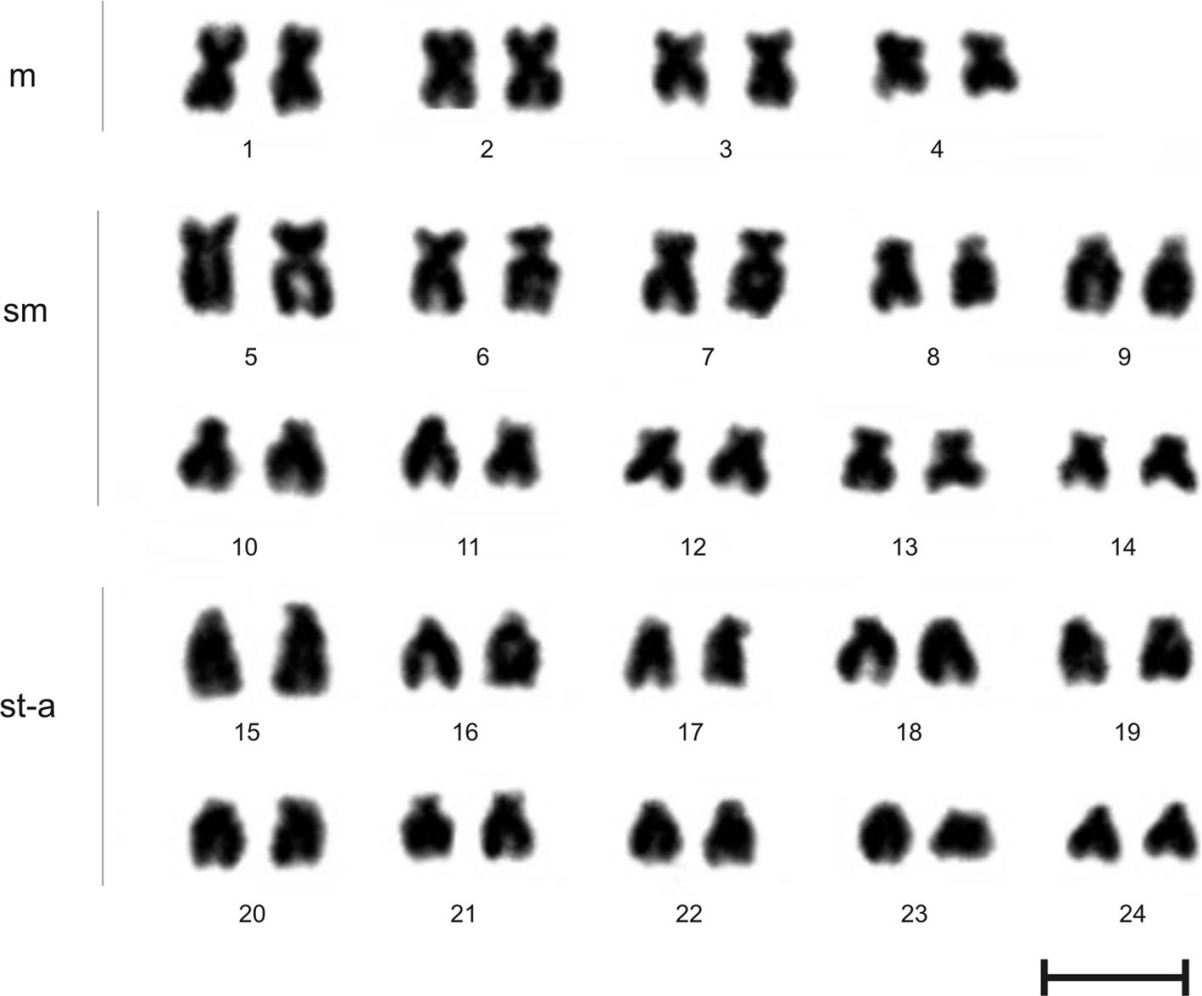
Karyotype of the European bitterling *R*. *amarus* based on Giemsa staining. *m* Metacentric chromosomes, *sm* submetacentric chromosomes, *st–a* subtelo-acrocentric chromosomes; *Bar* = 10 μm

Karyotype composed of 8 m, 20 sm and 20 st–a chromosomes (2n = 48 and NF = 76) was found in all investigated individuals (Fig. 2).

**Fig. 3:**
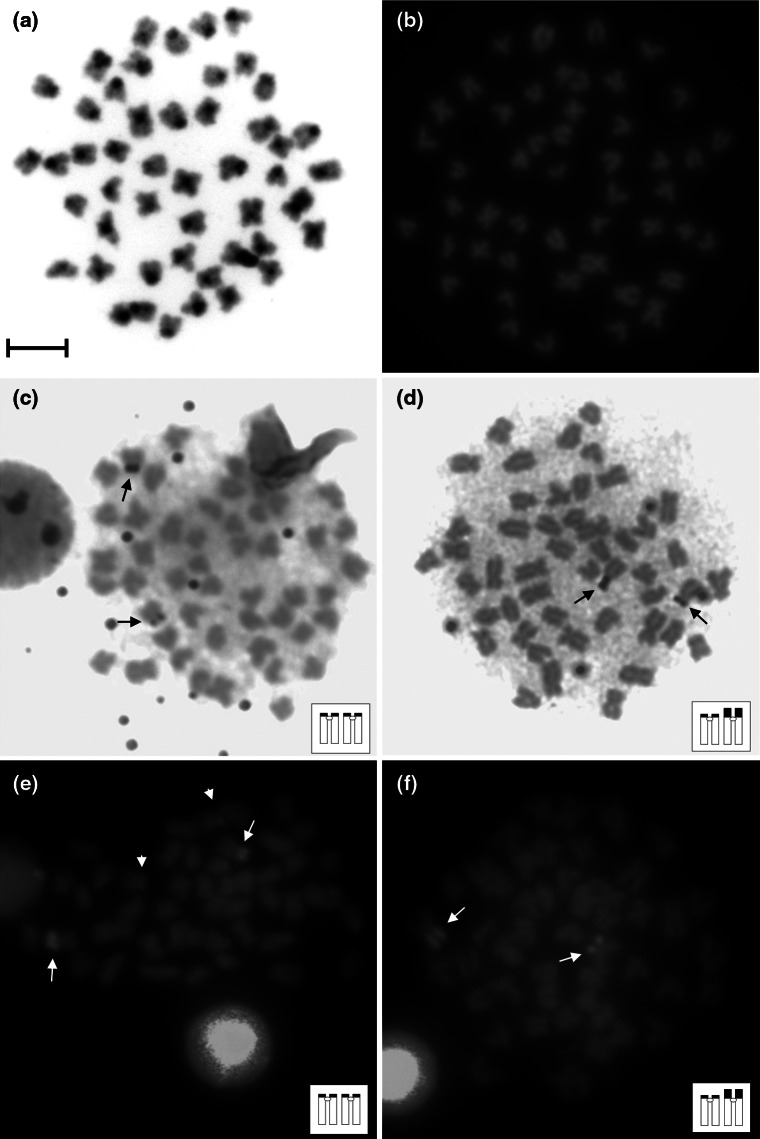
Chromosomes of the European bitterling *R*. *amarus* after C-banding (**a**), DAPI staining (**b**), Ag-NOR (**c**, **d**) and chromomycin A_3_ (CMA_3_) (**e**, **f**) staining. *Arrows* indicate Ag-NOR or GC-rich regions, respectively. *Arrowheads* indicate additional weak CMA_3_-positive signals. *Bar* = 10 μm

Blocks of the C-band positive heterochromatin were identified at the pericentromeric regions in most of the bitterling chromosomes. Moreover, short arms of some of the bitterling chromosomes were entirely built with the heterochromatin (Fig. 3a).

After Ag-NOR staining, one sm pair with NORs located at the short (p) arms was found in the karyotype of the European bitterling (Fig. 3c). Additionally, NOR bearing chromosomes exhibited length polymorphism related to the variation of the Ag-NOR size. Two isoforms of the NOR bearing chromosome were observed: chromosome with a short p-arm (s) and chromosome with a long p-arm (l). Among studied specimens, two cytotypes, namely ss and sl (in two females from the Bug River) were found (Fig. 3c–d).

Chromomycin A_3_ staining revealed GC-rich chromatic blocks on the p-arms of the two NOR bearing sm chromosomes (Fig. 3e–f). In some cases, one or two additional discrete CMA_3_ positive signals were also visible on the small a chromosomes (Fig. 3e). Size polymorphism of the CMA_3_-positive sites strictly corresponded with the length polymorphisms of the Ag-NOR segments (Fig. 3e–f). NOR related CMA_3_ -positive sites were negatively stained with DAPI fluorochrome. No DAPI positive signals were detected in any of the bitterling chromosomes (Fig. 3b).

**Fig. 4:**
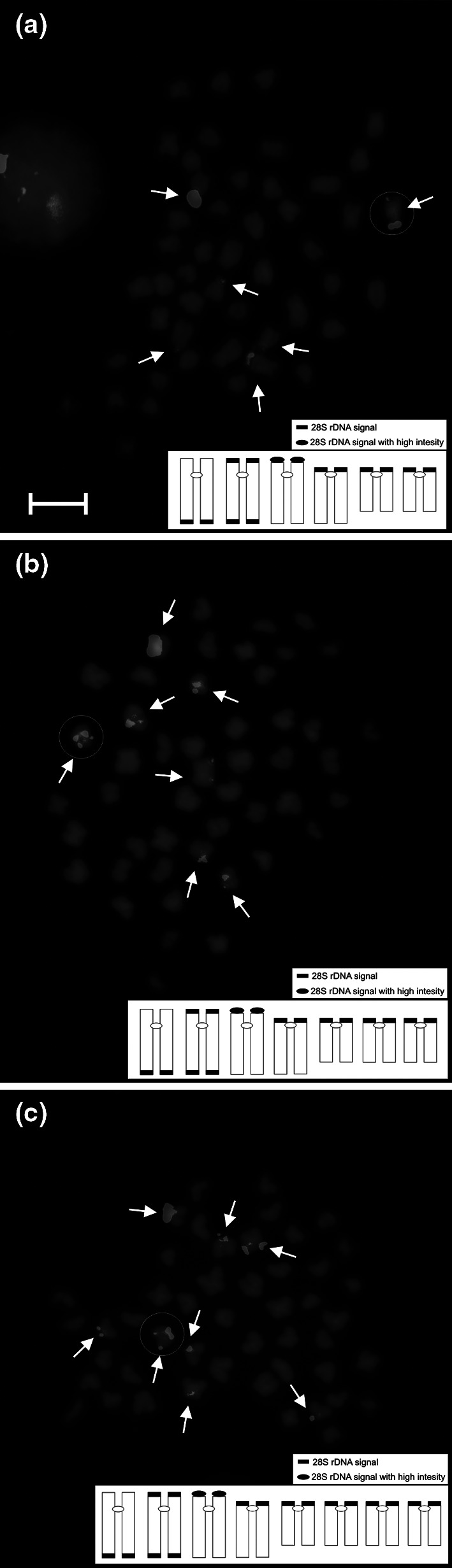
Chromosomes of the European bitterling *R.* *amarus* after FISH with 28S rDNA probe. *Arrows* indicate signals after hybridization. *White circle* indicates a chromosome with rDNA signals on both short and long arms. *Bar* = 10 μm

After FISH with 28S rDNA probe, hybridization signals were observed on one or both arms of three sm chromosomes and p-arms of three (Fig. 4a), four (Fig. 4b) or five (Fig. 4c) a chromosomes. Fluorescent intensity and number of the fluorescent spots observed on the a chromosomes varied intra-individually. Three patterns of the distribution of the hybridization signals were proposed based on the number of a chromosomes with 28S rDNA fluorescent signals (Fig. 4).

**Fig. 5:**
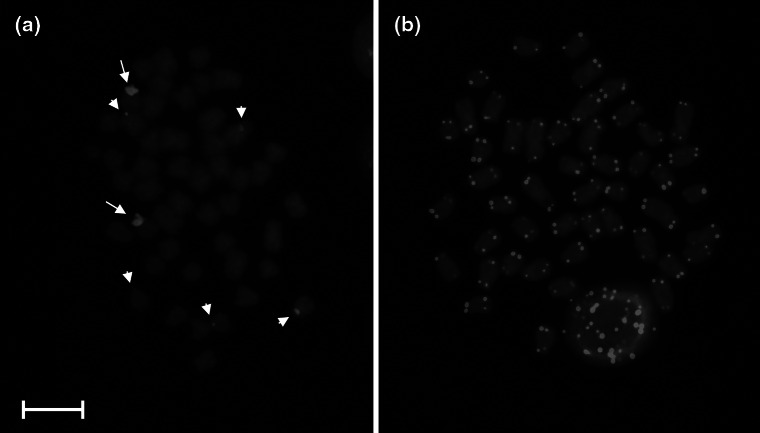
Chromosomes of the European bitterling *R.* *amarus* after PRINS with 5S rDNA probe (**a**), and PRINS with telomere probe (**b**). *Arrows* indicate strong signals while *arrowheads* weak signals after amplification. *Bar* = 10 μm

Two distinct hybridization signals overlapping p-arms of two st chromosomes were obtained in the course of PRINS labeling with 5S rDNA primers. Moreover, additional and discrete hybridization spots were randomly distributed among the bitterling st and sm chromosomes. Such additional signals were usually observed at the pericentromeric positions (Fig. 5a).

The telomeric repeats were localized at the ends of all bitterling chromosomes. We did not find any internally located telomeric DNA sequences (Fig. 5b).

## Discussion

Diploid chromosome numbers (2n) and chromosome arm numbers (NF) in the biterling species from three genera, *Acheilognathus*, *Rhodeus* and *Tanakia*, may vary from 42 to 48 and from 50 to 86, respectively (Hafez et al. 1978; Ueda 2007). Such diversity has been attributed to both Roberstonian and tandem fusions, chromosomal inversions, and some minor rearrangements involving heterochromatic regions (Ueda 2007). Although cytogenetic examination of *R. amarus* specimens from various European locations exhibited some differences in the karyotype formulae, diploid chromosome number was invariably 48 in all studied individuals (Table 1). Some discrepancies in the morphological description of the bitterling chromosomes could be due to the technical problems with equivocal identification of the small bitterling chromosomes as uni- or bi-armed.

**Table 1:** Summary of chromosome studies of the European bitterling *R.* *amarus*

Diploid chromosome number (2n) [Chromosome arm number (NF)]	Karyotype formulae	Ag-NOR	CMA3	Major rDNA (18S or 28S rDNA)	Minor rDNA (5S rDNA)	Telomeric DNA sequences	References
48 [82]	34 msm + 14 a	–	–	–	–	–	Sofradzija et al. (1975)
48 [80]	6 m + 26 sm + 4 st + 12 a	–	–	–	–	–	Bozhko et al. (1976)
48 [80]	6 m + 26 sm + 16 a	–	–	–	–	–	Meszaros and Kato (1976)
48 [86]	14 m + 24 sm + 10 st–a	–	–	–	–	–	Hafez et al. (1978)
48 [76]	8 m + 20 sm + 20 st	2 sm	2 sm	2 sm + 2–5 sm–a	–	–	Libertini et al. (2008)
48 [76]	8 m + 20 sm + 20 st–a	2 sm (p)	2 sm (p)	3 sm + 3–5 a	MC	TS	present paper

*2n* diploid chromosome number, *NF* chromosome arm number, *m* metacentric chromosomes, *sm* submetacentric chromosomes, *st* subtelocentric chromosomes, *a* acrocentric chromosomes, (p), size polymorphism, *TS* terminal sites in all chromosomes, *MC* (multichromosomal), two major and numerous smaller hybridization signals, *Ag-NOR* silver-stained nucleolus organizer region staining, *CMA*_*3*_ chromomycin A_3_ staining

In the bitterlings studied to date, pericentromeric regions of most or even all chromosomes are composed of the C-banded heterochromatin. Additional large heterochromatic blocks covering almost entire p-arms of some of the chromosomes similar to these described in the present paper are also observed in *Tanakia signifier, Rhodeus atremius fangi, Rhodeus ocellatus ocellatus*, among others (Ueda 2007). Moreover, in some specimens of *R*. *ocellatus* and in *Rhodeus atremius fangi*, interstitial C-bands resulted from the paracentric inversions and tandem fusions located on the long arms have been also reported (Ueda 2007).

A single chromosome pair with CMA_3_ positive Ag-NORs seems to be a major pattern in the European bitterling, even though additional small AgNO_3_ sites have been detected in a few specimens caught in Italy (Libertini et al. 2008). Two NOR-bearing chromosomes have been also described in *R. ocellatus ocellatus* as well as in *R. ocellatus kurumeus* (Sola et al. 2003). NOR-related length polymorphism similar to that described in the bitterlings here is a condition frequently observed in many Teleostean species (e.g. Kirtiklis et al. 2005). Lack of the homozygous phenotype “ll” among any European bitterlings studied to date (Libertini et al. 2008, present study) may be explained by a lethal condition of such cytotype (Porto-Foresti et al. 2007).

Variable copy numbers of rDNA sequences at different chromosomal locations may result in the variation of the hybridization signal intensity or even their numbers as too short rDNA arrays may be below resolution of the FISH technique. Such intraindividual variation regarding number of the 28S rDNA sites has been also reported in other cyprinids (Gromicho et al. 2005, Boroń et al. 2006). Clusters of the 28S rDNA sequences that are not co-localized with the AgNO_3_ positive sites in the European bitterlings might be inactive NORs, however it is more probable that such located rDNA sequences are incomplete 45S rDNA units including 28S rDNA sequences redistributed within the genome by the transposable or retrotransposable elements (Nakajima et al. 2012). One or two pairs of chromosomes with clusters of 5S rDNA sequences have been already described in the bitterling species. *R*. *ocellatus* similar to *R. amarus* has a single chromosome pair with 5S rDNA sequences (Kikuma et al. 1999; present paper). Multiple location of 5S rDNA sequences observed in *Acheilognathus tabira* subsp. seems to be more derivative state, as *A. tabira* show reduced chromosome number (2n = 44) (Inafuku et al. 2000). The tiny additional 5S PRINS signals observed at the pericentromeric regions of a few European bitterling chromosomes studied here might have derived from the regions built with the repetitive DNAs other than 5S rDNAs but amplifiable by the primers utilized for PRINS method. In *Hoplias malabaricus*, a tandemly repetitive centromeric DNA sequence share sequence similarities with the repeat units of the 5S rDNA (Martins et al. 2006). On the other hand, multichromosomal distribution of the minor rDNA clusters observed here might also appear in the course of the transposition (Zhang et al. 2008; Cioffi et al. 2010).

Despite similar karyotype characteristics, FISH with telomeric probe showed different distribution patterns of the hybridization signals in the European bitterling and the Japanese bitterling (*R*. *ocellatus kurumeus*). While telomeric DNA sequences in the European bitterling chromosomes were seen exclusively at the terminal positions, the Japanese bitterling exhibited also non-terminal telomeric signals that have been detected at the pericentromeric locations of 14–16 chromosomes (Sola et al. 2003). It is puzzling why bitterling species sharing the same karyotype characteristics exhibit various distribution patterns of the telomeric DNA sequences. It is conceivable that telomeric DNA sequences being relicts of the ancestral chromosome fusions may retain at the fusion sites in one species and experience successive loss and degeneration in another species (Meyne et al. 1990; Slijepcevic 1998; Ocalewicz 2013).

In conclusion, the data presented in this paper expanded our knowledge about cytogenetic characteristics of the European bitterlings. Karyotype of the specimens from the eastern lineage showed multiple location of the major rRNA genes, of which two coincided with the NOR regions. In turn, 5S rDNA sites were found on two chromosomes only. No internally located telomeric DNA sequences were observed in the studied fish.
